# Emergency obstructed hernia admissions over the COVID-19 national lockdowns: a regional review

**DOI:** 10.1308/rcsann.2024.0060

**Published:** 2025-10-01

**Authors:** A Thaventhiran, N Nazar, D Balasubramaniam, C Bailey

**Affiliations:** ^1^ Maidstone and Tunbridge Wells NHS Trust, UK; ^2^ Queen Mary University of London, UK

**Keywords:** Hernia, COVID-19, Acute abdomen, Obstruction, Surgery

## Abstract

**Introduction:**

Emergency obstructed hernias pose a significant clinical challenge and can lead to higher complication rates, prolonged recovery, bowel resection and recurrence. Early diagnosis, urgent surgical intervention and appropriate antimicrobial prophylaxis are key. Our study aimed to describe the impact of national lockdowns on emergency obstructed hernia admissions and the effect on patient demographics and compliance with recommendation rates. Should another lockdown be in place in the future this could help us prepare plans for appropriate care and service provision.

**Methods:**

Patient records for those who received emergency surgery on obstructed hernias at a single site during the three COVID-19 lockdown periods in England were reviewed and underwent thematic analysis. A temporal comparison was undertaken, and 105 patients met the inclusion criteria.

**Results:**

There were no significant differences in age, American Society of Anesthesiologists (ASA) grade and length of stay. The mean duration of symptoms was 1,307 ± 1,779 days in the lockdown group vs 215 ± 593 days in the control group (*p* < 0.005). Long-standing hernias were the primary reason for admission in the lockdown group compared with ‘other medical issues’ in the control group. Both the 7-day (7.5% vs 4.6%; *p* < 0.05) and 30-day (10.0% vs 7.7%; *p* < 0.05) re-admission rates were higher in the lockdown group than the control group.

**Conclusions:**

This study suggests that more patients presented in obstruction and as an emergency with long-standing hernias during the COVID-19 lockdown. We recommend enacting adequate postoperative follow-up to reduce higher rates of re-admission, and better patient education or discussions with primary care to ensure adequate forward referrals of hernias.

## Introduction

Abdominal wall hernias are a common surgical condition. Patients may present in an emergency with bowel obstruction, incarceration or strangulation. An obstructed or incarcerated hernia is one in which the contents of the hernia become irreducible and can be complicated by intestinal obstruction. Strangulation can occur because of a compromised blood supply. Such hernias pose a significant clinical challenge and can lead to higher complication rates, prolonged recovery, bowel resection and recurrence. Early diagnosis, urgent surgical intervention and appropriate antimicrobial prophylaxis are key. Evidence from clinical commissioning groups (CCGs) shows a rationing of the surgical management of abdominal wall hernias in different CCGs, where some of the prerequisites for surgery include pain and a history of incarceration.^1^ Obstructed abdominal wall hernias in the National Audit of Small Bowel Obstruction have shown poor outcomes.^2^

COVID-19 was categorised as a pandemic by the World Health Organization on 11 March 2020, and profoundly impacted healthcare services worldwide. Several changes to practice had to be made to deal with the rising number of emergency admissions secondary to COVID-19. In our centre, patients on waiting lists for elective surgery were impacted the most and saw a substantial delay in their treatment. However, emergency cases needed to be treated as a necessity, and there remains a lack of data on how emergency surgical diseases were affected by the pandemic and subsequent lockdowns.^3^ Our study aimed to identify differences in the presentation and demographics of obstructed abdominal wall hernias over lockdown compared with an identical period before the pandemic. We also looked at adherence to guidelines with respect to the management of obstructed hernias. This information could help in the preparation of plans for appropriate care and service provision should another lockdown be in place in the future.

### Objectives

Our aims were to look at the following parameters for patients admitted as an emergency with abdominal wall hernias: compliance with antibiotics for strangulated bowel or those requiring a bowel resection, compliance with consultant care for those with the highest mortality, 7-day and 30-day re-admission rates, demographic differences between those admitted during lockdown or in the control period, and differences in the history or presenting complaint between the lockdown and control groups.

## Methods

We performed this cross-sectional study based on the standards defined by documents from the World Society for Emergency Surgery (WSES), the Royal College of Surgeons of England (RCS England) and the British Hernia Society for emergency obstructed hernias and emergency repair of obstructed hernias.^4–6^ This study was registered with the local audit department (# 922) and with the National Research Ethics Service as an audit.

A single researcher reviewed clerking notes to perform a thematic analysis of 105 patients, looking at the patient’s history and identifying any particular themes that emerged to indicate what caused acute presentations.

The study involved a review of the records of patients who received emergency surgery on obstructed hernias at the Maidstone and Tunbridge Wells NHS Trust during three lockdown periods in England. A temporal comparison was made between the three lockdown periods and three comparable periods in the previous year (control). The three lockdowns were dated: 20 March 2020 to 15 June 2020, 5 November 2020 to 2 December 2020 and 21 December 2020 to 12 April 2021; the control groups were dated: 21 December 2018 to 12 April 2019, 20 March 2019 to 15 June 2019 and 5 November 2019 to 2 December 2019. International Statistical Classification of Diseases and Related Health Problems, 10th revision and Classification of Interventions and Procedures were used to extract a list of patients with emergency hernia admissions operated on during the defined periods.

**Figure 1:**
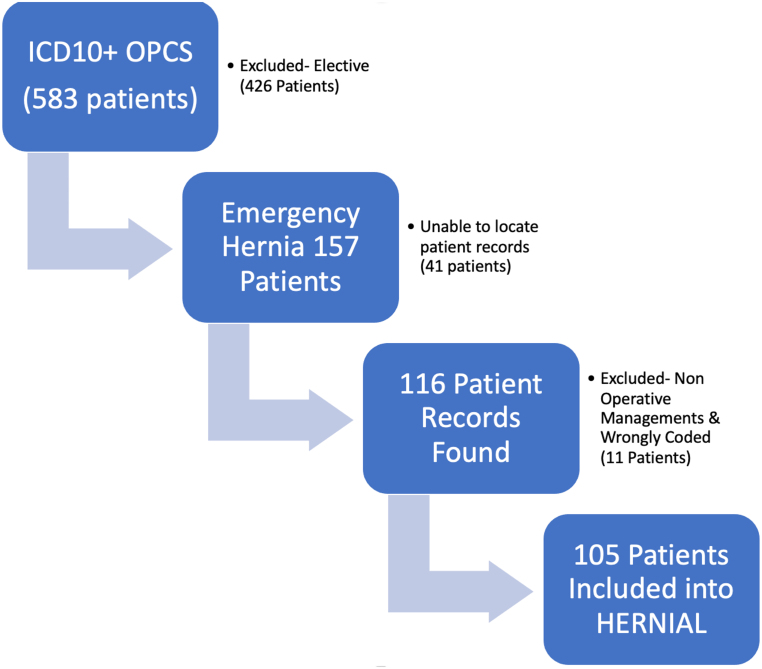
STROBE flow diagram depicting notes capture process

Of the 583 charts reviewed, patients with elective operations, those with no records available, those with non-operative management of hernias and incorrect coding were excluded from the study. A total of 105 patients met the inclusion criteria. A STROBE flow chart showing the number of patients identified, included and analysed during the study periods is given in Figure 1.

Four researchers retrospectively reviewed patients’ charts, surgical notes and discharge summaries to obtain metrics such as age, gender, ethnicity, American Society of Anesthesiologists (ASA) grade, grade of surgeon, use of mesh, imaging type, length of stay, duration of symptoms and antibiotic course length. Reasons for patient admissions were collected. Rates of strangulation, bowel resection, 7-day re-admission, 30-day re-admission, ASA grade ≥4, operated by consultant, and antibiotics given for 48h for bowel obstruction and/or bowel resection were also collected. High risk was defined as an ASA grade ≥4.

The aim of the study was to evaluate whether a nationwide lockdown (compared with baseline) influenced the rate of compliance to guidelines for 7-day and 30-day re-admission rates, antimicrobial prophylaxis for 48h for bowel obstruction and/or bowel resection, and compliance with consultant-delivered care for those with the highest mortality, and furthermore to determine whether lockdown influenced patient characteristics/demographics on admission and history of presenting complaint.

### Statistical analysis

Non-categorical data are presented as median and interquartile range. Categorical data are shown as a percentage of ‘*n*’ observations, where ‘*n*’ is the number of patients studied. Categorical data were analysed with a two-sided chi-squared test. All statistical tests and graphs were calculated, analysed and plotted on Prism 9 for MacOS (version 8.3.0). Thematic analysis of qualitative data was conducted using NVivo 14 (QSR International Pty Ltd, 2015) according to the principles of framework analysis. Patient notes were used to generate specific statements based on the clerking doctor’s documentation. Statements were then analysed to generate themes and codes.

## Results

A total of 586 patients with hernias were reviewed, 105 of whom were included. Some 426 patients had elective procedures and were excluded. Forty-one patients had no official records, and eleven patients had non-operative management or were wrongly coded; these patients were also excluded. Looking further into these eleven patients, two were admitted during the lockdown periods but absconded from the hospital before any operation could be carried out or reviewed by a consultant surgeon. Two were admitted with small bowel obstruction from an internal hernia, underwent a laparotomy and were coded incorrectly. Four (two in the lockdown group and two in the control group) were seen in the emergency department with their hernia but were listed for an elective repair. No documented complications were noted for these four patients. Three patients (two from the control group and one from the lockdown group) were admitted for medical issues unrelated to a pre-existing hernia. They were treated for that issue only and were not seen by the surgical team.

Patients were divided into two groups: those who received emergency surgery on obstructed hernias during lockdown and those who received emergency surgery on obstructed hernias before lockdown (control). The baseline demographics and characteristics of patients, including age, gender, ethnicity, ASA grade, grade of the surgeon, type of hernia, use of mesh, imaging type, length of stay, duration of symptoms and antibiotic course length are shown in Tables 1 and 2.

**Table 1:** Patient characteristics and demographics

	All	Control	Lockdown	*p*-value
	(*n* = 105)	(*n* = 65)	(*n* = 40)	
Male (%)	51.4	47.7	56.4	0.40
Age, years (sd)	65 (20)	68 (18)	78 (12)	0.14
Day of admission (%)				
Monday	16.2	13.8	17.9	0.44
Tuesday	18.1	16.9	17.9	0.85
Wednesday	18.1	15.4	20.5	0.27
Thursday	12.4	12.3	10.3	0.55
Friday	14.3	16.9	10.3	0.15
Saturday	11.4	13.8	7.7	0.15
Sunday	10.5	10.8	10.3	0.82
Grade of surgeon operating (%)				
Consultant	22.6	21.7	24.2	0.74
Staff grade and associate specialist	9.7	16.7	30.3	**0.03**
Specialist registrar	55.9	58.3	45.5	0.09
Mesh (%)	39.2	36.7	43.2	0.39
ASA (sd)	2.4 (0.9)	2.4 (0.1)	2.3 (0.8)	0.67
Length of stay, days (sd)	6 (6)	7 (7)	5 (4)	0.55
Duration of symptoms, days (sd)	562 (1207)	215 (593)	1307 (1779)	**0.0005**
Course of antibiotics length, days (sd)	2 (3)	3 (4)	1 (1)	0.12

ASA = American Society of Anesthesiologists' physical status classification system

**Table 2:** Types of hernias in patients

	All	Control	Lockdown	*p*-value
Type (%)				
Femoral	15	15	17	0.70
Incisional	17	10	31	**0.0002**
Inguinal	28	34	17	**0.0058**
Parastomal	4	4	3	0.70
Spigelian	3	3	0	0.08
Umbilical/para	25	25	26	0.87
Ventral	9	9	6	0.42
Imaging (%)	43	66	53	0.06
Type of imaging (%)				
Computed tomography	38	35	86	**<0.0001**
Ultrasound	24	23	10	**0.01**
X-ray	38	40	5	**<0.0001**
Contents (%)				
Appendix	1	2	0	0.16
Bladder	2	4	0	**0.04**
Fat	25	18	36	**0.003**
Large bowel	8	9	6	0.42
Omentum	13	13	12	0.96
Small bowel	51	55	46	0.20

There were no significant differences in age, ASA grade, length of stay, or antibiotic course length. A significant difference was noted in the duration of symptoms at admission between the two groups with *p* < 0.0005 (Table 1).

The most common type of hernia operated on during the lockdown period was incisional hernia (31%) compared with inguinal hernias (34%) in the control group. Of the patients in the lockdown group, 66.2% had some form of imaging compared with 52.5% in the control group, with computer tomography (CT) being the favoured imaging modality in the lockdown group vs x-ray in the control group (Table 2).

Long-standing hernias (32% of patients) were the primary reason for admission in the lockdown group compared with ‘other medical issues’ (30% of patients) in the control group (Table 3). The other medical pathologies included stroke, heart failure and acute atrial fibrillation. Home-based activities, new-onset hernias and other medical issues as reasons for admission were more prevalent in the control group.

**Table 3:** Patient reasons for admission

Patient reason for admission	Lockdown (%)	Control (%)	*p*-value
Home-based activities (exercises, DIY, etc.)	1	8.5	**0.009**
Long standing	32	16	**0.008**
New onset	5	9	0.268
Other medical issues highlighted	18	30	**0.047**

Both the 7-day and 30-day re-admission rates were higher in the lockdown group than the control group (Table 3). In the lockdown group, 3 of 40 (7.5%) patients were re-admitted within 7 days, and 4 of 40 (10%) were re-admitted within 30 days compared with 3 of 65 (4.6%) within 7 days and 5 of 65 (7.7%) within 30 days in the control group. The standard (<5% re-admission rate) was achieved only for the 7-day re-admission rate in the control group.

Guidelines dictate that patients deemed to be high risk (ASA≥4 or predicted mortality >5%) are operated on by a consultant surgeon and anaesthetist. Nine patients had predicted mortality >5% in the control group, of whom four (44%) received consultant-led care (Table 4). Consultant-led care was delivered for the one patient who was deemed to be high risk in the lockdown group. Antimicrobial prophylaxis should be given for a minimum of 48h in all patients with bowel obstruction and/or bowel resection to reduce the risk of infection. Twelve of 40 patients (30%) meeting these criteria received prophylaxis in the lockdown group compared with 25 of 65 patients (38.5%) in the control group (Table 4).

**Table 4:** Standards achieved in lockdown and control groups

Criteria	Standard to be achieved (%)	Lockdown (%)	Control (%)
Patients considered high risk (>5% mortality) should be operated on by a consultant surgeon and consultant anaesthetist	100	100	44
7-Day re-admission rate	<5	7.5	4.6
30-Day re-admission rate	<5	10.0	7.7
Antimicrobial prophylaxis for at least 48h in patients with bowel obstruction and/or bowel resection	100	30.0	38.5

## Discussion

To our knowledge, this study is one of the first to describe the impact of national lockdowns on emergency obstructed hernia admissions in the United Kingdom (UK) and the effect on patient demographics and compliance with recommendations rates. In our study, admissions over lockdown were lower than pre-pandemic levels, which is in line with what was seen nationwide for admissions for surgical emergencies.^7^

More patients presented with incisional hernias in the lockdown group than in the control group; it could be that patients with inguinal hernias were less likely to present over the lockdown period. Contrary to the belief that more presentations would be secondary to home-based activities (DIY or exercise), we note more patients presented later and with long-standing hernias. Only 1% of patients presented secondary to home-based activities. The mean duration of symptoms for the lockdown group prior to admission was 1,307 days compared with 215 in the control group (*p* < 0.005). This could be explained by hospital avoidance and difficulties in accessing primary care and subsequent referral.^8^ Avoidance and fear of hospitals likely stemmed from worrying media reports of hospitals being overwhelmed by COVID-19, alongside issues such as shortages of equipment and oxygen.^9,10^ However, only indirect evidence exists and the exact reasoning for this increased duration of symptoms remains uncertain.

In our centre, there was little change to practice over the lockdown period. However, we saw poor re-admission rates and compliance with guidelines compared with both the standard and our practice prior to lockdown. This may be attributed to several reasons. People with comorbidities were advised to ‘shield’ and avoid face-to-face contact. The mean age in our lockdown group was higher at 78 years (sd 12) compared with 68 years (sd 18) in the control group. This could mean that high-risk patients were presenting later with more complicated diseases, and this resulted in poorer outcomes, as suggested by the higher re-admission rates seen. Furthermore, some evidence over the pandemic does point towards hospital avoidance leading to delayed admission and, therefore, more advanced disease.^7^ Two patients even self-discharged before being reviewed by a surgeon.

Studies point towards a greater length of stay for patients admitted to general surgery over the lockdown periods, which is hypothesised to be related to delayed admission and increased disease severity.^8,9^ However, in our study, the mean length of stay for the lockdown group was lower than that for the control group. Hospital capacity across the UK was near maximum, which meant that attempts could have been made to discharge the seemingly least sick individuals. Such suboptimal discharges could explain the higher re-admission rates seen here.

None of the patients re-admitted in the lockdown group tested positive for COVID-19, either in the initial presentation or during the re-admission to hospital. The main reason for re-admission in the two groups was surgical site infection, with one patient suffering from an early recurrence in the control group.

Several factors can affect surgical wound healing and postoperative infection rates for hernia repair. These include wound contamination (bacterial burden), timing and type of surgery, and mesh. The WSES recommends at least 48h antimicrobial prophylaxis for all patients with strangulation and/or bowel resection.^4^ In our study, we found poor compliance with antibiotic prescribing in both the lockdown (30%) and control groups (38.5%). The reasons for this are unclear and perhaps led to higher re-admission rates across both groups compared with the required standard (<5%). CT scanning was the primary type of imaging over lockdown, with 86% of patients receiving CT scans compared with just 35% in our control group. This may be due to a desire to determine whether the hernia definitely required surgical intervention in the lockdown group because taking patients to theatre when their COVID-19 status was unknown or uncertain was avoided if possible during that time.

RCS England recommends that patients who are predicted to have >5% mortality (ASA ≥4) should be operated on with a consultant present.^5^ Our groups showed good consultant cover for the sickest patients, with 100% compliance over lockdown. This could be because of our centre’s practice of risk-assessing all emergency surgical patients and using ASA as the standard tool for risk assessment. This allowed patients to be matched with the appropriate seniority and level of care.

### Study limitations

The main limitation of this study was the small number of patients across a single region, which may impact the ability to draw broader conclusions and also not be representative of other regions with different patient demographics. The heterogeneous group of patients seen and a single coder for thematic analysis are further limitations to this study. The design of the study is inherently at risk of chronological bias, namely the hospital policy for imaging changed from plain x-ray to CT scanning for patients presenting with suspected abdominal pathology between the two study periods.

## Conclusions

Unique and unprecedented changes to clinical practice have been seen worldwide in response to the COVID-19 pandemic. With respect to emergency obstructed hernias, better compliance with antibiotic guidelines is key, alongside enacting adequate follow-up to reduce the slightly high rates of re-admission seen. Discussions in local governance meetings may help tackle compliance issues and discussions with primary care to ensure adequate forward referrals of hernias and better patient education.

Furthermore, our data suggest more patients presenting in obstruction and emergency with long-standing hernias, which differs from what was traditionally thought. Although several factors may have influenced such presentations over lockdown, it is worth revisiting in the post-pandemic era. Long-standing hernias tend to be managed conservatively, but our study has perhaps shown that we may have to rethink management pathways and treatment for this group of patients, who are often elderly with multiple comorbidities. Such patients tend to have poorer outcomes, which may be exacerbated by issues surrounding the rationing of care alongside the delays and wait times seen because of COVID-19.
